# The bHLH Transcription Factor PhbHLH121 Regulates Response to Iron Deficiency in *Petunia hybrida*

**DOI:** 10.3390/plants13233429

**Published:** 2024-12-06

**Authors:** Liru Pan, Chengcheng Huang, Ruiling Li, Yanbang Li

**Affiliations:** School of Agriculture and Biology, Shanghai Jiao Tong University, Shanghai 200240, China; pan-liru@sjtu.edu.cn (L.P.); huangchengcheng@sjtu.edu.cn (C.H.); liruiling@sjtu.edu.cn (R.L.)

**Keywords:** *Petunia hybrida*, basic-helix-loop-helix, bHLH121, iron deficiency

## Abstract

Iron (Fe) is an essential micronutrient for plants. Due to the low Fe bioavailability in cultivated soils, Fe deficiency is a widespread agricultural problem. In this study, we present the functional characterization of a petunia (*Petunia hybrida*) basic-helix-loop-helix transcription factor PhbHLH121 in response to Fe shortage. Real-time PCR revealed that the expression of *PhbHLH121* in petunia roots and shoots was downregulated under Fe-limited conditions. CRISPR/Cas9-edited *phbhlh121* mutant plants were generated to investigate the functions of PhbHLH121 in petunia. Loss-of-function of PhbHLH121 enhanced petunia tolerance to Fe deficiency. Further investigations revealed that the expression level of several structural genes involved in Fe uptake in petunia, such as *IRT1* and *FRO2*, was higher in *phbhlh121* mutants compared to that in wild-type under Fe-limited conditions, and the expression level of several genes involved in Fe storage and Fe transport, such as *VTL2*, *FERs* and *ZIF1*, was lower in *phbhlh121* mutants compared to that in wild-type under Fe-deficient conditions. Yeast one-hybrid assays revealed that PhbHLH121 binds to the G-box element in the promoter of genes involved in Fe homeostasis. Yeast two-hybrid assays revealed that PhbHLH121 interacts with petunia bHLH IVc proteins. Taken together, PhbHLH121 plays an important role in the Fe deficiency response in petunia.

## 1. Introduction

Iron (Fe) is an essential micronutrient for plants. It functions as a co-factor for several important enzymes and plays a crucial role in many fundamental biological processes, such as chlorophyll biosynthesis, photosynthesis, nitrogen fixation, and hormone biosynthesis [1,2]. Although Fe is one of the most abundant elements in most soils, it frequently exists in the form of insoluble ferric oxides, which are poorly available to plants, especially in alkaline soil [3]. About 30% of the world’s cultivated soils are calcareous. Therefore, Fe deficiency is common for plants, leading to poor nutritional quality and reduced crop yield [2].

To overcome low-Fe conditions, higher plants have developed two major strategies for Fe uptake: reduction strategy (strategy I) and chelation-based (strategy II). Non-graminaceous plants use the reduction strategy. In Arabidopsis, H^+^-ATPases AHA2 secretes protons into the soil, increasing the solubility of Fe [4]. The FERRIC REDUCTION OXIDASE2 (FRO2) catalyzes the reduction of Fe^3+^ to Fe^2+^, and then Fe^2+^ is transported into the root via IRON-REGULATED TRANSPORTER1 (IRT1) [5,6]. Graminaceous plants mainly use strategy II to cope with Fe deficiency. Graminaceous plants release phytosiderophores, which belong to the mugineic acid (MA) family to obtain the Fe^3+^ phytosiderophore complex. Then, the resulting complex is transported into plant roots by YELLOW STRIPE 1 (YS1) and YELLOW STRIPE 1-like (YSL) transporters [7,8].

The expression of genes involved in Fe homeostasis, including *FRO2* and *IRT1*, is tightly controlled in plants under Fe-deficient conditions. The basic-helix-loop-helix (bHLH) transcription factors (TFs) play key roles in Fe deficiency response. Among them, the FER-like iron deficiency-induced transcription factor (FIT) is a crucial regulator in inducing the expression of Fe uptake genes of strategy I under Fe-limited conditions [9,10]. In Arabidopsis, FIT interacts with bHLH Ib TFs (bHLH38, bHLH39, bHLH100, and bHLH101) to activate the expression of *IRT1* and *FRO2*. Thus, the Arabidopsis *fit* mutants exhibit severe Fe deficiency symptoms, such as reduced iron content, chlorosis and are lethal under iron deficiency [9,11,12].

In contrast to FIT, Arabidopsis bHLH47/OPEYE (PYE) was originally described as a transcriptional repressor in the Fe deficiency responses [13,14]. PYE interacts with bHLH105/ILR3 and represses the expression of genes involved in Fe transport (*nicotianamine synthase 4*), Fe storage (*FER1*, *FER3*, *FER4,* and *VTL2*), and Fe assimilation (*NEET*) [13,14]. The loss of PYE function causes the inhibition of root growth under Fe-deficient conditions in Arabidopsis. And the Fe content in the roots and shoots of *pye* mutants is significantly higher than that in wild-type (WT) plants [13].

The key transcriptional regulator upstream of FIT and PYE in Arabidopsis has been studied recently [15,16,17,18]. Arabidopsis bHLH121 interacts with ILR3 and acts as a direct transcriptional regulator of genes involved in Fe homeostasis, including *PYE* and bHLH Ib FTs [15,16,18]. Although the expression of *FIT* was also reduced in *bhlh121* mutant in response to Fe deficiency, *FIT* is not a direct target of bHLH121 [16,18]. *bhlh121* loss-of-function mutants have decreased tolerance to Fe deficiency [15,16,18]. In contrast, bHLH11, the closest homolog of bHLH121 in Arabidopsis, acts as a negative regulator of Fe homeostasis [19,20]. The expression of *IRT1*, *FRO2,* and *bHLH38/39/100/101* was upregulated in the *bhlh11* mutant under control, Fe excess, and Fe-deficient conditions. Loss-of-function of bHLH11 displays enhanced sensitivity to excess Fe, but not Fe deficiency [19].

*Petunia hybrida* (Solanaceae) is the world’s most popular bedding plant and already has a long history of use as a model organism in plant molecular biology and genetics research [21,22]. Petunia, which takes strategy I for Fe uptake, is an Fe-inefficient plant. When grown on neutral and high pH conditions, petunia exhibit Fe deficiency symptoms, such as chlorosis and growth retardation [23]. Moreover, the Fe content in the flower also influences the color of the flower and petal senescence, and both of them are important traits for ornamental plants [24,25,26,27]. However, the Fe homeostasis network remains largely unknown in petunia. In the present study, we characterized the roles of petunia PhbHLH121 in Fe homeostasis. The CRISPR/Cas9-edited *phbhlh121* petunia mutants exhibit enhanced tolerance to Fe deficiency. The interaction studies showed that PhbHLH121 interacts with four petunia bHLH transcriptional factors closely related to Arabidopsis bHLH34, bHLH104, bHLH105, and bHLH115. Further analysis revealed that the expression of petunia Fe homeostasis genes, such as *FIT*, *PYE*, *IRT1*, *FRO2*, *ZIF1*, and *VTL2,* was affected by PhbHLH121 under Fe-deficient conditions.

## 2. Results

### 2.1. PhbHLH121 Encodes a bHLH Protein

**Figure 1:**
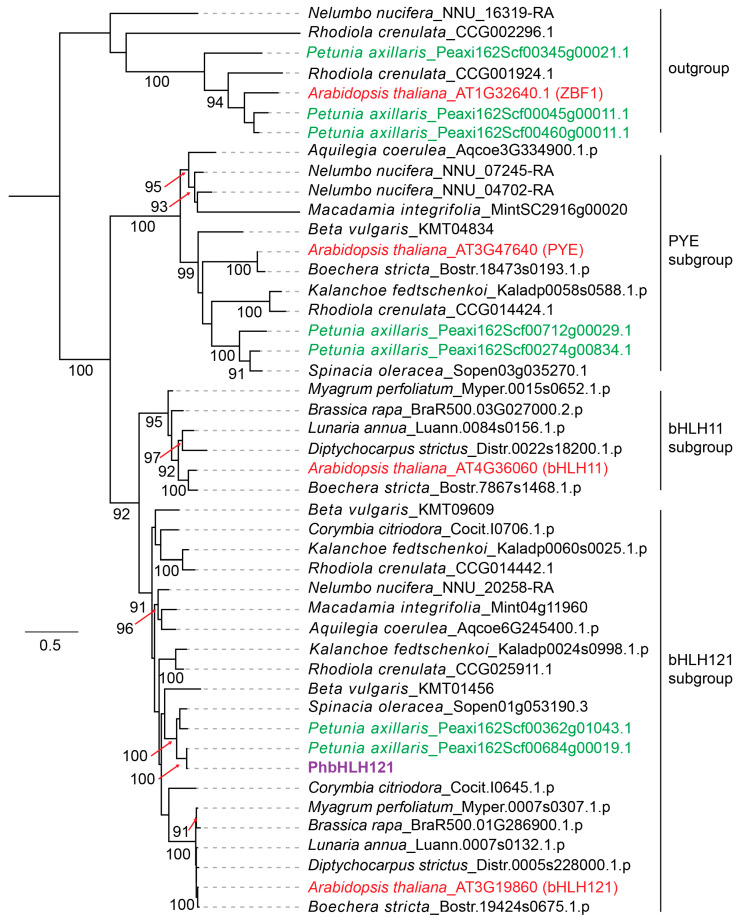
Maximum-likelihood phylogenies of bHLH proteins from different species. Phylogenetic tree was inferred using IQ-TREE under the JTT+G4 model. Branch support is indicated on the tree branches as percentage of 1000 bootstraps if ≥90%. PhbHLH121 are marked in purple color. *Petunia axillaris* bHLH proteins are marked in green color. Arabidopsis bHLH proteins are marked in red color. ZBF1 proteins are set as the outgroup.

The full-length CDS of *PhbHLH121* was cloned from roots of *Petunia hybrida*. *PhbHLH121* encodes a protein of 339 amino acids containing a typical bHLH domain (Figure S1). Multiple sequence alignment showed that PhbHLH121 shares 57%, 45%, and 38% identity with Arabidopsis bHLH121, bHLH11, and PYE, respectively (Figure S1). These three Arabidopsis proteins belong to the bHLH IVb subfamily [28]. Furthermore, we constructed a maximum-likelihood tree of several bHLH IVb proteins from different plant species. ZBF1, a repressor of blue light-mediated photomorphogenic growth, belongs to the bHLH IIIe subfamily [28,29] and was used as the outgroup. The phylogenetic tree showed that petunia PhbHLH121 and Arabidopsis AtbHLH121 clustered into the same subgroup, suggesting that PhbHLH121 may be involved in the regulation of Fe deficiency response (Figure 1).

### 2.2. Expression Pattern and Subcellular Localization of PhbHLH121

**Figure 2:**
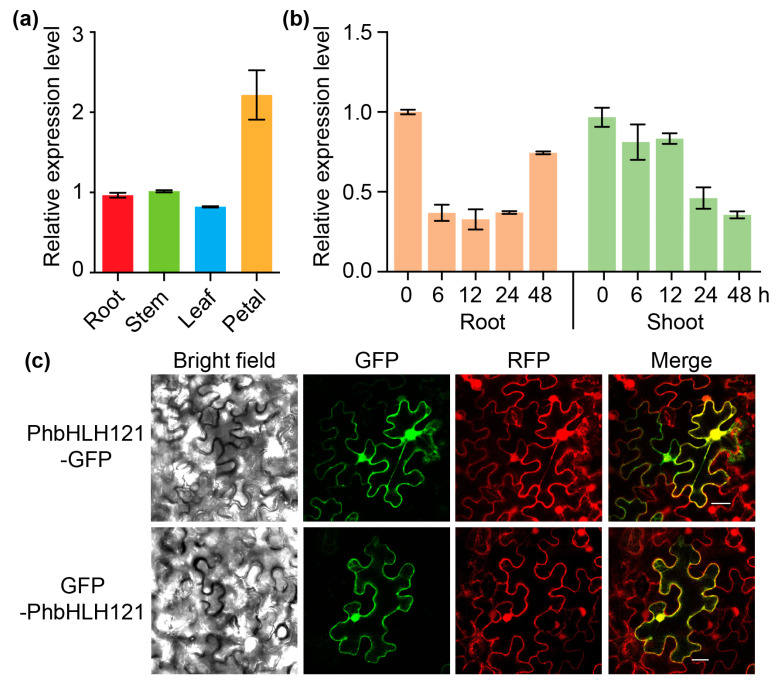
Expression pattern and subcellular localization of PhbHLH121. (**a**) Expression level of *PhbHLH121* in different organs of 8-week-old petunia. Values are means ± SD (n = 3). *EF1α* was used as reference gene. Expression was normalized to that in roots. (**b**) Expression pattern of *PhbHLH121* in petunia seedlings after exposure to Fe-deficient condition. Values are means ± SD (n = 3). *EF1α* was used as reference gene. Expression was normalized to that at 0 h. (**c**) Subcellular localization of PhbHLH121-GFP, GFP-PhbHLH121, and free RFP in tobacco leaves. Scan bar = 20 µm.

The expression of *PhbHLH121* in petunia was analyzed using real-time RT-PCR. We found *PhbHLH121* expressed in the root, stem, leaf, and petal, and slightly higher in the petal compared to the other organs (Figure 2a). To investigate the response of *PhbHLH121* to Fe status, 5-day-old petunia seedlings grown on half-strength Murashige and Skoog (MS) medium were transferred to half-strength MS media supplied with 300 μM ferrozine to effectively deplete Fe (−Fe) [30]. Six hours after Fe deficiency treatment, the expression level of *PhbHLH121* was downregulated in roots, and the expression level was also downregulated in shoots 24 h after the treatment (Figure 2b).

To study the subcellular localization of PhbHLH121, we generated *35S*:*PhbHLH121*-*GFP* (GFP fused to C-terminus of PhbHLH121) and *35S*:*GFP*-*PhbHLH121* (GFP fused to N-terminus of PhbHLH121) constructs and transiently co-expressed these two constructs with free Red Fluorescent Protein (RFP) in *Nicotiana benthamiana* leaves by agroinfection. The green fluorescence signal was mainly present in the nucleus and cytoplasm, which co-localized with free RFP (Figure 2c), indicating that PhbHLH121 was localized in the nucleus and cytoplasm.

### 2.3. PhbHLH121 Interacts with bHLH IVc TFs

**Figure 3:**
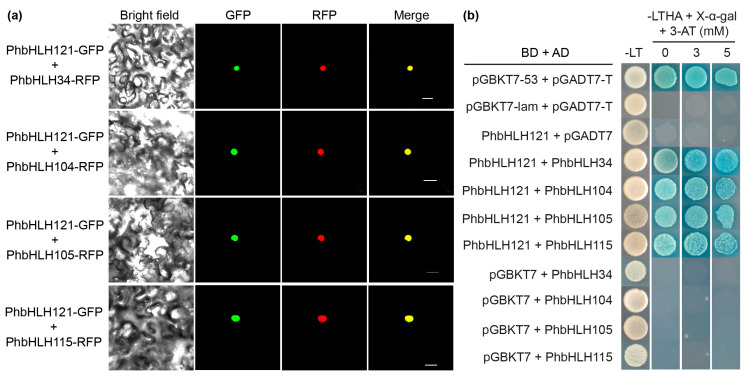
bHLH IVc TFs interact with PhbHLH121. (**a**) Subcellular localization of PhbHLH121 in the presence of petunia bHLH IVc TFs in tobacco leaves. PhbHLH121-GFP was co-transformed with PhbHLH34-RFP, PhbHLH104-RFP, PhbHLH105-RFP, or PhbHLH115-RFP into tobacco cells. GFP and RFP signals were visualized under a confocal microscope. Scan bar = 20 µm; (**b**) Y2H assays. bHLH IVc TFs were fused with the GAL4 activation domain (AD) and PhbHLH121 with the GAL4 DNA binding domain (BD). Yeast co-transformed with different BD and AD plasmid combinations was spotted on selective medium lacking leucine, tryptophan, adenine, and histidine (-LTHA) and contained a different concentration of 3-amino-1,2,4-triazole (3-AT).

Arabidopsis bHLH11 and bHLH121 have been observed in both nucleus and cytoplasm and their nuclear accumulation has been shown to be promoted by bHLH IVc transcription factors (TFs) [15,16,19]. Next, we investigated whether petunia bHLH IVc TFs could affect the subcellular localization of PhbHLH121. PhbHLH121-GFP was co-expressed with bHLH IVc TFs fused to RFP in tobacco leaves. The GFP signal was mainly observed in the nucleus (Figure 3a), indicating that bHLH IVc TFs affect the subcellular localization of PhbHLH121. To further investigate whether PhbHLH121 interacts with bHLH IVc TFs, we performed GAL4-based yeast two-hybrid assays. When co-expressed PhbHLH121-fused GAL4 DNA binding domain (BD) with the GAL4 DNA binding domain fused to bHLH IVc TFs in yeast, yeast cells grew on the selective medium, indicating that PhbHLH121 and petunia bHLH IVc TFs physically interact in yeast (Figure 3b).

### 2.4. Loss of PhbHLH121 Function Alters Response to Iron Deficiency

**Figure 4:**
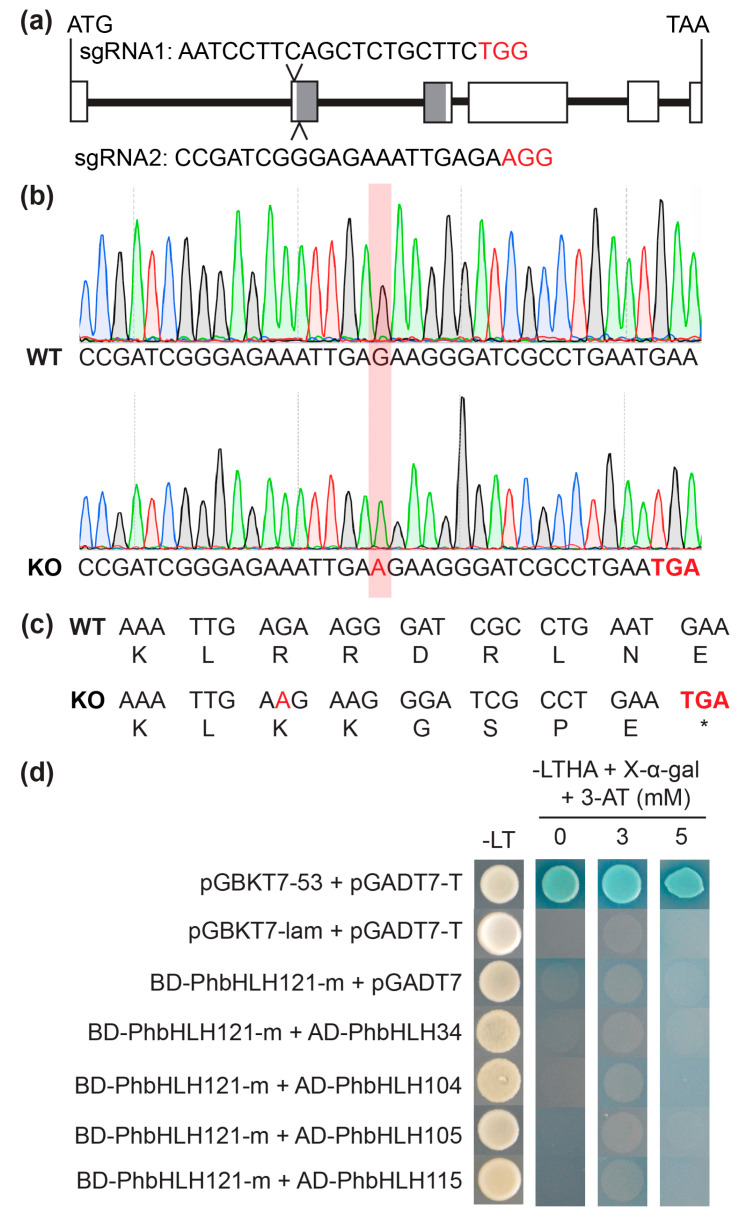
*phbhlh121* CRISPR/cas9-edited mutation. (**a**) Schematic representation of *PhbHLH121* gene and localization of the two RNA guides. Gray boxes indicate the bHLH conserved domain; (**b**) Sanger sequencing results of *PhbHLH121* in wild-type (WT) and KO plants. The red box indicates the localization of “A” nucleotide insertion. (**c**) Protein sequence comparison of WT and *phbhlh121*-*ko* mutation. The premature stop codon was marked in bold red text. The asterisk represents the end of translation; (**d**) Y2H assays. PhbHLH34, PhbHLH104, PhbHLH105, and PhbHLH115 were fused with the GAL4 activation domain (AD), and *phbhlh121*-*ko* mutation (PhbHLH121-m) with the GAL4 DNA binding domain (BD). Yeast co-transformed with different BD and AD plasmid combinations was spotted on selective medium lacking leucine, tryptophan, adenine, and histidine (-LTHA) and contained a different concentration of 3-AT.

To investigate whether PhbHLH121 plays a role in Fe homeostasis in petunia, we generated *phbhlh121* knockout (KO) mutants using the clustered regularly interspaced short palindromic repeat (CRISPR)-associated protein9 (Cas9) genome editing system (CRISPR/Cas9). We designed the CRISPR/Cas9 construct containing two gRNA sequences (Figure 4a). One homozygous mutant with single nucleotide insertion in exon 2 was generated (Figure 4b), which caused a frameshift and premature stop codons in the N-terminal region of the bHLH conserved domain (Figure 4c). The *phbhlh121*-*ko* mutation (*PhbHLH121*-*m*) was amplified and cloned into the pGBKT7 vector. Yeast cells co-expressed BD-PhbHLH121-m and AD-bHLH IVc were unable to grow on the selective medium, indicating that the *phbhlh121*-*ko* mutation lost the ability to interact with bHLH IVc TFs in yeast cells (Figure 4d).

**Figure 5:**
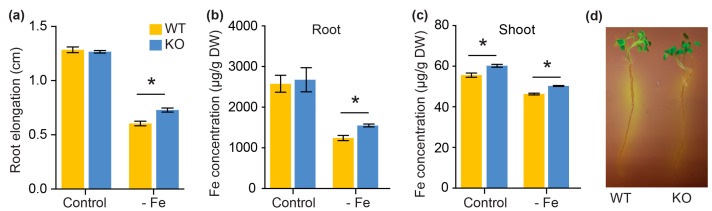
Iron deficiency response of *phbhlh121* mutants. (**a**) Root elongation of 7-day-old petunia seedlings exposed to a medium supplied with different Fe concentrations in the last 2 days. Values are means ± SD (n = 20); (**b**,**c**) Fe concentration in roots (**b**) and shoots (**c**) of petunia plants exposed to Fe-deficient conditions for 2 d. Values are means ± SD (24 plants in total per line). DW, dry weight; (**d**) rhizosphere acidification of 5-day-old WT and KO plants exposed to Fe-deficient media for 3 d. Then, plants were transferred to agar plates containing bromocresol purple for 18 h. Acidification is indicated by the yellow color around the roots. The asterisks indicate that the values are significantly different from the corresponding WT value by Student’s *t* test (*p* < 0.05).

After 5 days on half-strength MS medium, seedlings were transferred to Fe-sufficient medium, Fe-excess medium, and Fe-deficient medium for 2 days, and the root elongation during exposure was measured. The root elongation was inhibited similarly by excess Fe in WT and KO mutants (Figure S2). In contrast, when grown on -Fe medium, the root elongation in WT was significantly shorter than that in KO mutants (Figure 5a). Furthermore, elemental analysis showed that the Fe concentration in roots and shoots of KO plants was significantly higher than that of WT (Figure 5b,c). Additionally, we investigated whether loss-of-function of PhbHLH121 impairs Fe deficiency-induced rhizosphere acidification; we transferred 5-day-old plants grown on the half-strength MS medium to the Fe-deficient medium for 3 days and then placed them on an agar plate containing bromocresol purple for 18 h. The typical acidification feature of yellow color was observed around WT roots; however, the KO plants barely showed discoloration around the roots (Figure 5d). These results indicate that loss-of-function of PhbHLH121 impairs the Fe deficiency response.

### 2.5. Loss-of-Function of PhbHLH121 Disrupted Expression of Fe Deficiency-Responsive Genes

**Figure 6:**
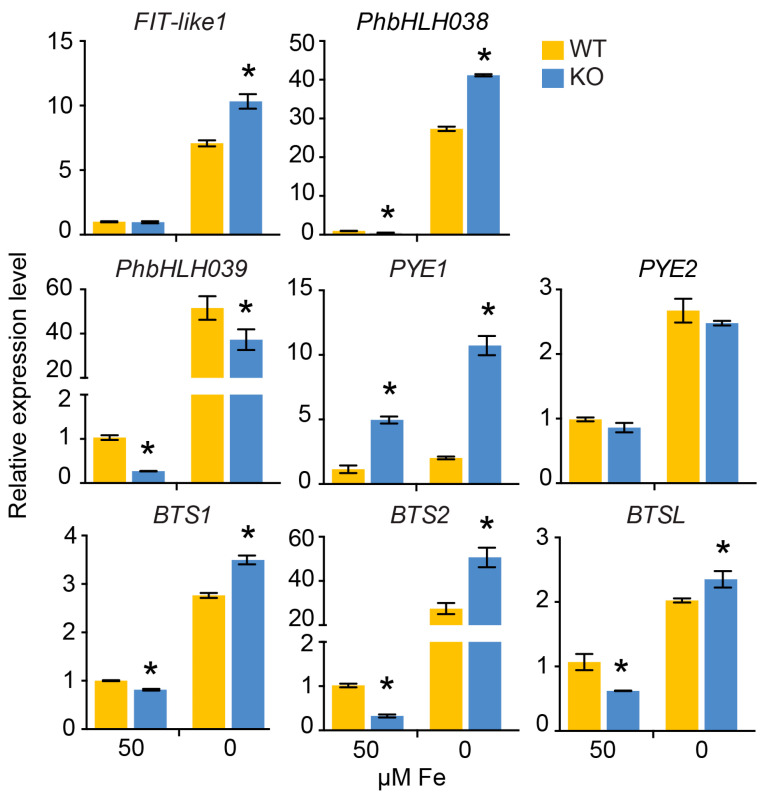
Expression of several genes involved in Fe homeostasis transcriptional regulatory network in KO plants. Relative expression was determined by RT-qPCR in 1-week-old petunia seedlings grown on Fe-sufficient or Fe-deficient media. Values are means ± SD (n = 3). *EF1α* was used as reference gene. Expression was normalized to that in WT seedlings grown on the Fe-sufficient medium. The asterisks indicate that the values are significantly different from the corresponding WT value by Student’s *t* test (*p* < 0.05).

To further investigate the effect of PhbHLH121 on Fe homeostasis genes, real-time PCR was carried out in 7-day-old seedlings grown on an Fe-sufficient medium and an Fe-deficient medium. First, the relative expression of several transcriptional regulatory proteins was examined. The expression of AtFIT homolog, *FIT-like1*, was upregulated in KO plants under Fe deficiency. Petunia PhbHLH038 and PhbHLH039 are homologs of Arabidopsis bHLH38, bHLH39, bHLH100, and bHLH101 [31]. The expression of *PhbHLH038* was higher in the KO plants than in the WT plants under Fe deficiency, whereas the expression of *PhbHLH039* was lower in the KO plants than in the WT plants. Two PYPEYE (PYE) homologs were found in petunia; the expression of *PYE1* was higher in the KO mutants than in WT regardless of Fe status, whereas no difference was observed for *PYE2*. The transcriptional level of E3 ubiquitin ligase BTS1, BTS2, and BTSL1 was higher in KO mutants compared to WT under Fe-limited conditions (Figure 6).

**Figure 7:**
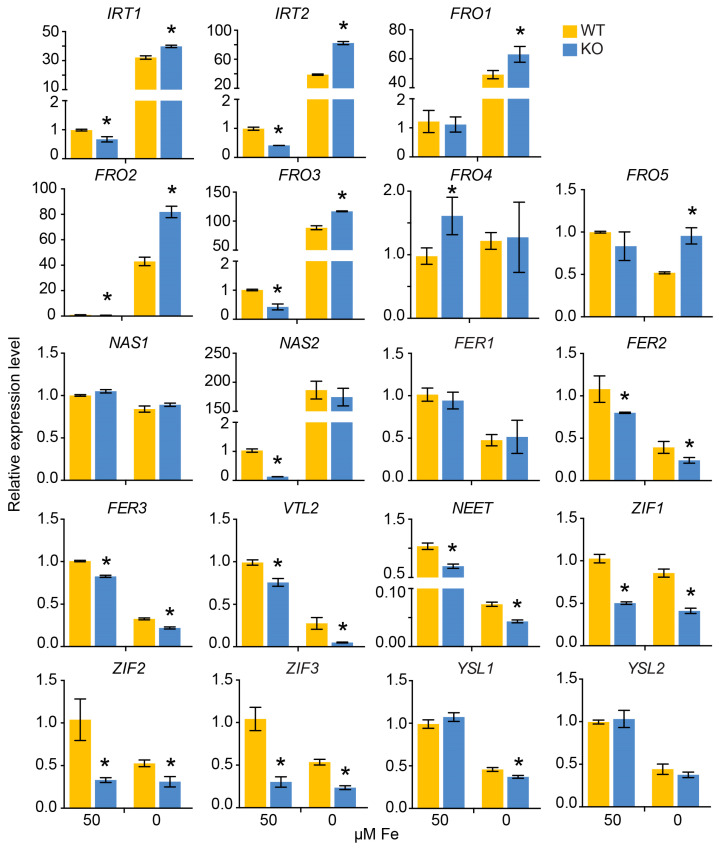
Expression of several structural genes involved in Fe homeostasis in KO plants. Relative expression was determined by RT-qPCR in 1-week-old petunia seedlings grown on Fe-sufficient or Fe-deficient media. *EF1α* was used as reference gene. Expression was normalized to that in WT seedlings grown on Fe-sufficient medium. Values are means ± SD (n = 3). The asterisks indicate that the values are significantly different from the corresponding WT value by Student’s *t* test (*p* < 0.05).

Then, the relative expression of structural genes involved in Fe homeostasis was examined. The expression of AtIRT1 homologs in petunia, *IRT1* and *IRT2*, was significantly upregulated in *phbhlh121* mutants compared to WT in response to Fe deficiency, especially for *IRT2* (Figure 7). The petunia genome encodes five AtFRO1/2/3 homologs. Petunia *FRO1*, *FRO2*, *FRO3*, and *FRO5* were expressed at higher levels in *phbhlh121* mutants under Fe deficiency than in the WT. In contrast, the expression level of genes involved in Fe storage, including *FERs* and *VTL2*, was significantly downregulated in *phbhlh121* mutants compared to WT in response to Fe deficiency. In particular, the expression of *VTL2* was reduced approximately 5-fold in *phbhlh121* mutants compared to WT under Fe-limited conditions (Figure 7). The expression level of *NEET*, which is involved in Fe assimilation, was also reduced in KO plants compared to WT under Fe-limited conditions. Several genes involved in Fe^2+^-nicotianamine transport, such as AtYSL1 homologs and AtZIF1 homologs, was also downregulated in KO plant under Fe deficiency (Figure 7). The expression of *NAS1* and *NAS2* was unaffected by loss-of-function of PhbHLH121 in response to Fe deficiency.

### 2.6. PhbHLH121 Binds to the G-Box Element in the Promoter

**Figure 8:**
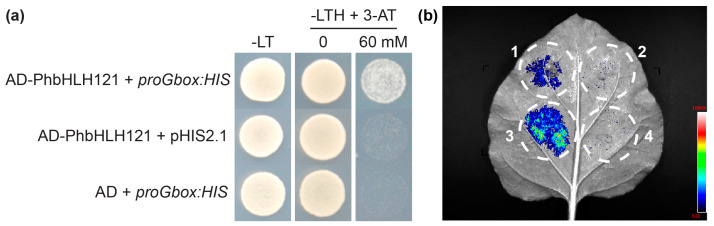
PhbHLH121 binds to the G-box element. (**a**) The interaction of PhbHLH121 with G-boxes as shown by yeast one-hybrid assay. Yeast cells that co-transformed with different AD and *pBait*:*His* plasmid combinations were spotted on the selective medium lacking leucine, tryptophan, and histidine (-LTH) and containing a different concentration of 3-AT. (**b**) Transient expression analyses confirming transactivation of *PhbHLH039* by PhbHLH121. Circle 1 indicates EV+ *proPhbHLH039*:*LUC*. Circle 2 indicates EV+ pGreenII 0800-LUC. Circle 3 indicates PhbHLH121 + *proPhbHLH039*:*LUC*. Circle 4 indicates PhbHLH121 + pGreenII 0800-LUC.

In Arabidopsis, bHLH121 binds to the G-box elements in the promoter of Fe-responsive genes [18]. In petunia, several Fe-responsive genes contain G-box elements in the promoter region (Figure S3). We then carried out a yeast one-hybrid assay to assess the interaction of PhbHLH121 with G-box element. Yeast cells harboring the AD-PhbHLH121 and *proGbox*:*HIS* grew on the selective medium lacking leucine, tryptophan, and histidine (-LTH), and containing 60 µM 3-AT. In contrast, yeast cells that co-expressed the control vectors could not survive on the selection medium (Figure 8a). These results suggest that PhbHLH121 binds to the G-box element. In Arabidopsis, bHLH039 is a direct target of bHLH121 [16,18]. Next, we tested the effects of PhbHLH121 on the expression of *PhbHLH039* using the dual-luciferase (LUC) reporter system. The 2000 bp *PhbHLH039* promoter was cloned and fused to luciferase in the pGreenII 0800-LUC vector. In luminescence imaging assays, the tobacco leaves co-expressing PhbHLH121 and *proPhbHLH039*:*LUC* showed strong luminescence intensity, whereas weak or no visible luminescence signals were observed in the control (Figure 8b). These results suggest that PhbHLH121 activates the expression of *PhbHLH039*.

## 3. Discussion

The low Fe bioavailability in cultivated soils inhibits plant growth and reduces the quality of plant products. To cope with Fe shortage, plants have developed strategies to maintain Fe homeostasis through absorption, transport, distribution, and storage [32]. In this study, we show that petunia PhbHLH121 interacts with petunia bHLH IVc TFs and regulates the expression of several genes involved in Fe homeostasis. Loss-of-function of PhbHLH121 display enhanced tolerance to Fe deficiency.

PhbHLH121 belongs to the bHLH Ib subgroup, together with Arabidopsis AtbHLH11 and AtbHLH121. However, the functions of these proteins are different. AtbHLH11 has been identified as a negative TF that regulates the expression of several genes involved in Fe homeostasis, including *IRT1*, *FRO2*, *bHLH38*, *bHLH39,* and *bHLH100*. The *atbhlh11* mutants displayed enhanced sensitivity to excess Fe [19]. In contrast, *atbhlh121* loss-of-function mutants displayed severe defects in response to Fe deficiency [15,16,18]. AtbHLH121 works as a transcriptional activator of key genes involved in Fe homeostasis, such as *IRT1*, *FRO2*, *FIT*, *bHLH38*, *bHLH39*, *bHLH100,* and *PYE*. In this study, we show that petunia *phbhlh121* loss-of-function mutants displayed longer roots and increased Fe concentration in mutants compared to WT under Fe-limited conditions (Figure 5). This could be due to the mis-regulation of genes involved in Fe uptake, Fe transport, and Fe storage in *phbhlh121* mutants.

Transcriptional analysis revealed that petunia *IRT1*, *IRT2*, *FRO1*, *FRO2,* and *FRO3* were upregulated in WT plants in response to iron deficiency, and they were upregulated even more in *phbhlh121* mutants under Fe-limited conditions, which is consistent with the increased expression of *FIT-like1* and *PhbHLH038* in *phbhlh121* mutants (Figure 6). Therefore, our results indicate that PhbHLH121 regulates Fe uptake most likely through the FIT-dependent pathway. Y1H and LUC experiments indicated that PhbHLH121 binds to the G-box element in the promoter region and activates the expression of *PhbHLH039*. However, the expression of *PhbHLH038* was reduced in *phbhlh121* mutants compared to WT under Fe-limited conditions. It is possible that other transcription factors may be involved in this regulatory process, but this remains to be elucidated.

Plants store iron under Fe-sufficient conditions and release it when plants are exposed to Fe-limited conditions [13]. Two major storage strategies for Fe are sequestration into vacuoles and ferritin [2]. In Arabidopsis, PYE and ILR3 play important roles in regulating Fe storage and transport. PYE interacts with ILR3 to repress the expression of genes involved in Fe storage and transport, such as *NAS4*, *ZIF1*, *VTL2*, *FER1*, *FER3,* and *FER4* [13,14]. The Arabidopsis *pye-1* mutants exhibited Fe deficiency symptoms, such as chlorosis and inhibition of root growth [13]. PYE is a direct target of AtbHLH121, and the expression level of *PYE* was reduced in Arabidopsis *atbhlh121* mutants compared to that in WT under Fe-limited conditions. Interestingly, petunia *PYE1* expression is strongly increased in *phbhlh121* mutants compared to that in WT under Fe-limited conditions (Figure 6), indicating that PhbHLH121 may play a role in regulating Fe storage.

Given its redox activity, iron must be bound by chelators to be translocated effectively without causing damaging redox reactions. In plants, nicotianamine (NA) is a vital chelator for Fe homeostasis. NA is synthesized from S-adenosyl methionine by nicotianamine synthase (NAS). The Arabidopsis genome encodes four members of the NAS family, among which *NAS4* is a direct target of PYE [13]. The expression of *NAS4* was upregulated in *pye-1* mutants under Fe-limited conditions [13]. Unlike that of Arabidopsis, the petunia genome encodes only two NAS proteins: NAS1 and NAS2 [31]. The expression of *NAS1* and *NAS2* was unaffected by the loss-of-function of PhbHLH121 under Fe-limited conditions (Figure 7). *ZIF1*, a direct target of PYE in Arabidopsis, encodes a vacuolar membrane-localized major facilitator superfamily (MFS) transporter and is upregulated by iron deficiency in *pye-1* mutants compared to that in WT. ZIF1 is most likely to act as a transporter of free NA from the cytosol into the vacuole. The overexpression of *ZIF1* in Arabidopsis resulted in enhanced sensitivity to Fe deficiency [33]. The petunia genome encodes three ZIF1 homologs, all of which are downregulated in *phbhlh121* mutants under Fe-limited conditions. *VTL2* (*VACUOLAR IRON TRANSPORTER-LIKE 2*) encodes an Fe transporter that plays a role in transferring cytoplasmic Fe into vacuoles. Its expression was strongly increased in *pye-1* mutants under Fe-limited conditions [14,34]. A recent study has shown that AtbHLH121 functions as a repressor in the regulation of *VTL2* expression [18]. However, *VTL2* expression was decreased in petunia *phblh121* mutants under Fe-limited conditions. Together, the expression of *VTL2*, *ZIF1*, *ZIF2*, and *ZIF3* is reduced in *phbhlh121* mutants compared to that in WT under Fe-limited conditions, indicating that the Fe storage in the vacuole may be inhibited in *phbhlh121* mutants in response to Fe deficiency.

Ferritin is a ubiquitous protein for the transient storage of Fe and is able to buffer Fe to maintain Fe homeostasis [35]. The Arabidopsis genome encodes four ferritin proteins, FER1, FER2, FER3, and FER4. *FER1*, *FER3,* and *FER4* are mainly expressed in vegetative tissues, and FER2 is mainly expressed in seeds [36,37]. Recent studies have demonstrated that FER1, FER3, and FER4 are direct targets of bHLH121 and ILR3 [14,18]. The expression of *FERs* is strongly induced in response to excess Fe to avoid free iron-induced production of reactive oxygen species (ROS) [37]. After exposure to Fe-limited conditions, the expression of *AtFER1*, *AtFER3,* and *AtFER4* was significantly downregulated [14,17]. In petunia, three *FER* genes were identified, and their expression was downregulated under Fe-limited conditions. The expression levels of *PhFER2* and *PhFER3* were lower in *phbhlh121* mutants compared to that in WT under Fe-limited conditions, indicating that petunia PhbHLH121 may play a role in maintaining Fe levels by regulating the expression of *FERs*. Together, the expression of petunia *FER2*, *FER3*, *VTL2*, and three *ZIFs* was reduced in the *phbhlh121* mutant compared to WT in response to Fe shortage. These changes may result in decreased Fe storage and the release of stored Fe for the use of Fe-containing proteins.

Our work reveals that PhbHLH121 interacts with bHLH IVc TFs and plays roles in Fe uptake and Fe storage. In future research, the transcriptional regulatory network involving *PhbHLH121* in Fe homeostasis needs to be further explored.

## 4. Materials and Methods

### 4.1. Plant Material and Growth Conditions

*Petunia hybrida* (cv. Mitchell; W115) seeds were sterilized with 1:10 (*v*/*v*) diluted bleach for 10 min, then washed with H_2_O three times, and placed in 4 °C for 2 days. Then, seeds were sown on solid half-strength Murashige and Skoog (MS) medium. For the hydroponic experiment, 14-day-old seedlings were transferred to modified half-strength Hoagland’s nutrient solution: 3 mM KNO_3_, 2 mM Ca(NO_3_)_2_, 1 mM K_2_HPO_4_, 0.5 mM MgSO_4_, 20 µM FeEDDHA, 50 µM KCl, 25 µM H_3_BO_3_, 2 µM ZnSO_4_, 2 µM MnSO_4_, 0.2 µM CuSO_4_, and 0.5 µM (NH_4_)_6_Mo_7_O_24_. The pH was buffered at 5.5. The nutrient solution was renewed once a week. Petunia was grown in a climate room (temperature around 25 °C, with the photon flux density at plant level 120 µmoles m^−2^s^−1^, 14 h per day, and air humidity (RH) around 75%).

### 4.2. RNA Isolation and Quantitative Real-Time PCR

Total RNA was isolated from petunia tissues using RNAprep pure Plant Kit (TIANGEN, Beijing, China) according to the manufacturer’s instructions. The first strand cDNA was synthesized from 2.5 μg of total RNA using the HiScript III 1st Strand cDNA Synthesis Kit (Vazyme, Nanjing, China). Quantitative RT-PCR was performed on a Bio-Rad CFX384 real-time system (Bio-Rad, Hercules, CA, USA) using a SYBR^®^ Green Master Mix Kit (Vazyme, Nanjing, China). *EF1α* was used as the reference gene. The primers are listed in Table S1.

### 4.3. Cloning

The coding region of *PhbHLH121* was retrieved from the *Petunia axillaris* genome (v1.6.2) database [22], and gene-specific primers (Table S2) were designed to amplify the coding region from the roots cDNA of *Petunia hybrida* (line W115) using PrimeSTAR^®^ Max DNA Polymerase (R045Q, Takara, Beijing, China). Purified *PhbHLH121* CDS was cloned into multiple vectors to make functional plasmids for several purposes using a seamless cloning kit (Sangon Biotech, Shanghai, China). The coding sequence of petunia *PhbHLH34*, *PhbHLH104*, *PhbHLH105*, and *PhbHLH115* was obtained by blasting the sequence of Arabidopsis AtbHLH34, AtbHLH104, AtbHLH105, and AtbHLH115 in the *Petunia axillaris* genome (v1.6.2) database [22]. By using the same procedure as for *PhbHLH121*, the coding sequence of *PhbHLH34*, *PhbHLH104*, *PhbHLH105*, and *PhbHLH115* were amplified and cloned into multiple vectors. The promoter sequences of the PhbHLH039 gene were extracted from the *Petunia axillaris* genome (v1.6.2) database, and amplified from W115 gDNA with PrimeSTAR^®^ Max DNA Polymerase (R045Q, Takara, Beijing, China).

### 4.4. Multiple Sequence Alignment and Phylogenetic Analysis

The amino acid sequences of bHLH121 homologs in different plant species were obtained by using the protein sequences of petunia PhbHLH121, Arabidopsis AtbHLH121, Arabidopsis AtbHLH11, and Arabidopsis PYE as query sequence in BLASTp searches against annotated protein sequences from the Phytozome database v13 (https://phytozome.jgi.doe.gov/pz/portal.html, accessed on 1 August 2024). Best hits for each protein were selected for phylogenetic analysis. Multiple sequence alignment was carried out by MAFFT (v7.475) using the L-INS-i algorithm [38]. For the phylogenetic analysis, BMGE was used for the alignment curation [39]. The best-fit model for the maximum-likelihood tree building was selected with ModelFinder in IQ-TREE (v1.6.12) based on the Bayesian information criterion [40]. Then, the phylogeny was generated using IQ-tree (v1.6.12) using ultrafast bootstrap approximation (1000 replicates) for branch support [40,41]. Phylogenetic trees were visualized with iTOL (https://itol.embl.de/, accessed on 10 August 2024).

### 4.5. Subcellular Localization

*PhbHLH121* coding sequence was amplified from W115 cDNA by PCR using primers shown in Table S2 and recombined into pCAMBIA1300 to obtain *35S*:*PhbHLH121*-*GFP* and *35S*:*GFP*-*PhbHLH121* constructs. *PhbHLH34*, *PhbHLH104*, *PhbHLH105,* and *PhbHLH115* were amplified from W115 cDNAs and recombined into pCAMBIA1300 to obtain *35S*:*Gene*-*RFP* constructs. All constructs were each introduced into *A*. *tumefaciens* strain *GV3101*. After growth in Luria–Bertani liquid medium, *Agrobacterium* cells were harvested and resuspended in infiltration buffer (0.2 mM acetosyringone, 10 mM MgCl_2_, and 20 mM MES, pH 5.8) to OD_600_ = 0.6–0.8. Equal volumes of various combinations of *Agrobacterium* cells were mixed and co-infiltrated into *Nicotiana benthamiana* leaves with a needleless syringe as described previously [42]. Two days after the infiltration, epidermal cells were observed under a Leica microscope (Nussloch, Germany).

### 4.6. Yeast Two-Hybrid Assay

The coding sequences of *PhbHLH121* and *phbhlh121* mutation were amplified from cDNA of WT and KO, respectively. Then, the PCR products were recombined into pGBDT7. *PhbHLH34*, *PhbHLH104*, *PhbHLH105,* and *PhbHLH115* were amplified from W115 cDNAs by PCR using primers shown in Table S2 and recombined into pGADT7. Yeast two-hybrid assays were performed as described previously [43].

### 4.7. Generation of CRISPR/Cas9-Edited phbhlh121

Constructs for CRISPR/Cas9 mutagenesis were performed as previously described [44].

### 4.8. Fe Tolerance Evaluation

Fe tolerance evaluation was performed as described before [45,46]. Briefly, WT and KO petunia seedlings were grown on half-strength MS medium for 5 days, then seedlings were transferred to Fe-sufficient medium (50 µM Fe(III)-EDTA), Fe excess medium (300 µM Fe(III)-EDTA), and Fe-deficient medium (300 µM Ferrozine) for 2 days. Root elongation during exposure was measured.

### 4.9. Element Analysis

Fourteen-day-old petunia seedlings grown on half-strength MS medium were transferred to modified half-strength Hoagland’s nutrient solution. After three weeks in hydroponics, the plants were exposed to nutrient solution with different Fe concentrations (0 and 20 µM Fe) for 2 days. After the exposure, roots and shoots were collected for the element analysis. The experiments were performed with three replicates (24 plants in total). Samples were digested in H_2_O_2_/HNO_3_ (1:1, *v*/*v*) after drying at 70 °C for 48 h before measuring dry weight. Minerals were analyzed using inductively coupled plasma optical emission spectrometry (ICP-OES; Optima 8000, PerkinElmer, Waltham, MA, USA).

### 4.10. Rhizosphere Acidification Assay

Rhizosphere acidification was performed as previously described [47]. Briefly, petunia seeds were germinated and grown on half-strength MS medium for 4 days and then seedlings were transferred to Fe-limited medium for 3 days. Then, seedlings were transferred to a 1% agar plate containing 0.006% bromocresol purple and 0.2 mM CaSO_4_ (pH 6.5) for 18 h.

### 4.11. Yeast One-Hybrid Assay

The coding sequence of *PhbHLH121* was amplified from W115 cDNAs and recombined into the pGADT7 vector. For bait construction, the G-box element, repeated three times, was integrated into the pHIS2.1 vector to generate p*roGbox*:*HIS*. Different AD and pBait:His plasmid combinations were transformed into the Y187 strain. The interaction was tested on the selective medium lacking leucine, tryptophan, and histidine (-LTH) and containing 60 mM 3-AT. The primers used in this product are listed in Table S2.

### 4.12. Dual-Luciferase Reporter Assays

The promoter of *PhbHLH039* (2 kb) was amplified from W115 genomic DNA and cloned into the pGreenII 0800-LUC vector to generate the reporter construct. The effector and reporter constructs were co-introduced into *N. benthamiana* leaves. The luminescence of firefly luciferase was detected as described previously [48].

## Data Availability

All data supporting the findings of this study are available in the paper and the Supplementary Materials.

## Supplementary Materials

The following supporting information can be downloaded at: https://www.mdpi.com/article/10.3390/plants13233429/s1, Figure S1: Multiple sequence alignment of PhbHLH121, AtbHLH11, AtbHLH121, and PYE. Red box indicates the conserved bHLH domain; Figure S2: Root elongation of 7-day-old petunia seedlings exposed to medium supplied with excess Fe in the last 2 days; Figure S3: Promoter structure diagrams for several genes involved in Fe homeostasis; Table S1: List of primers used for real-time PCR in this study; Table S2: List of primers used for plasmid construction.
