# Supplementary material for: The bHLH Transcription Factor PhbHLH121 Regulates Response to Iron Deficiency in Petunia hybrida
Source: Plants (Basel). 2024 Dec 6;13(23):3429. doi: 10.3390/plants13233429 (PMC11644503; doi:10.3390/plants13233429)
Supplement: Supplementary file 1 [file plants-13-03429-s001.zip › plants-3232042-supplementary.pdf]

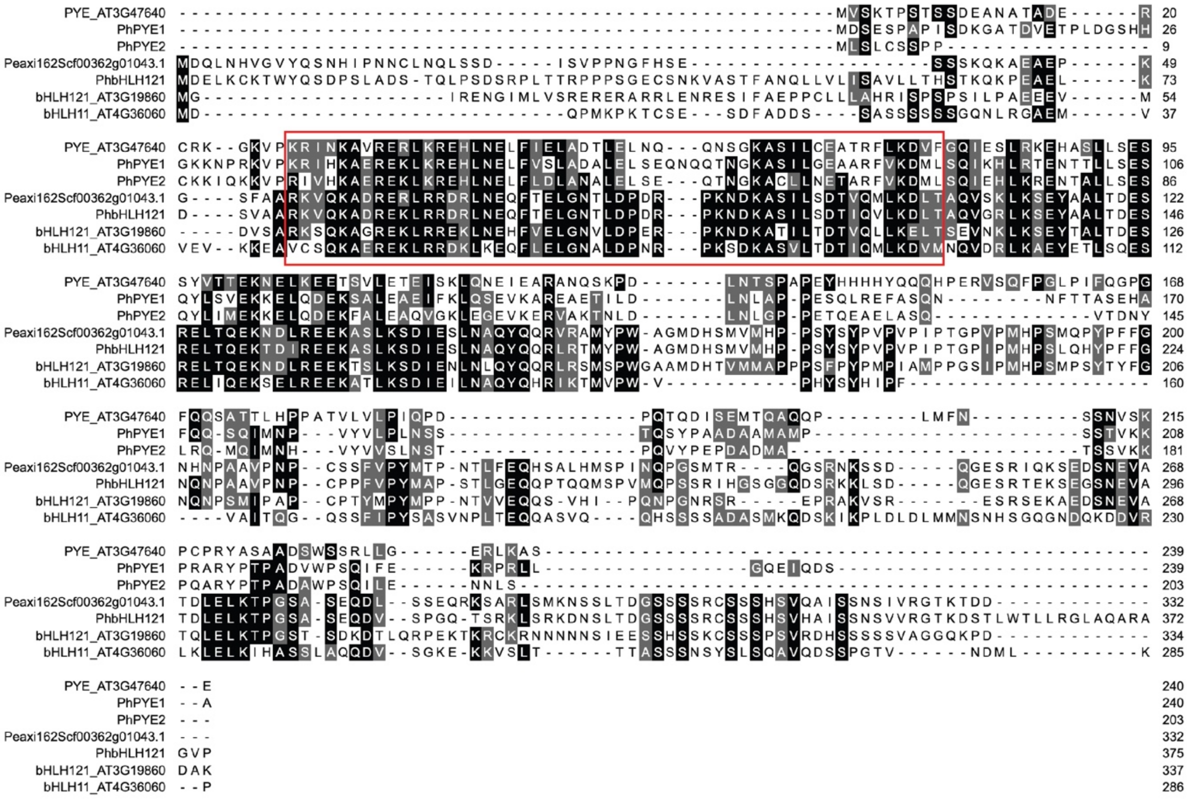

Figure S1. Multiple sequence alignment of PhbHLH IVb proteins. Red box indicates the conserved bHLH domain.

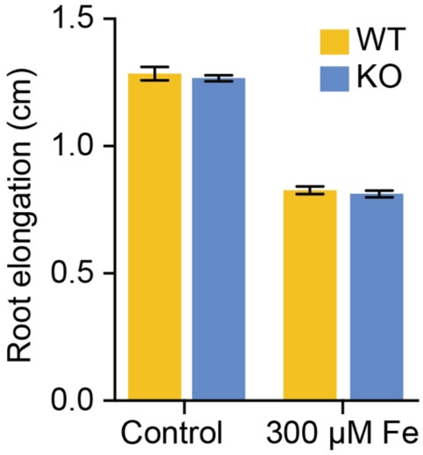

Figure S2. Root elongation of 7-day-old petunia seedlings exposed to medium supplied with excess Fe in the last 2 days. Values are means ± SD (n=20).

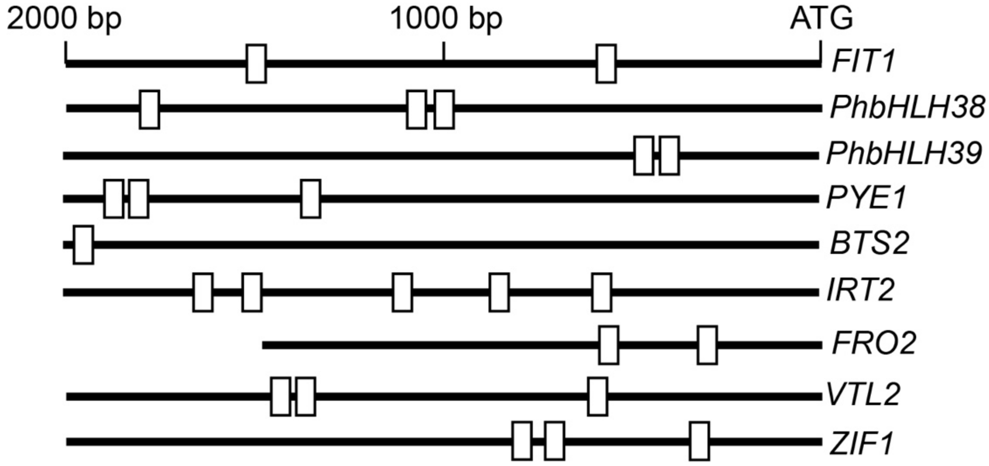

Figure S3. Promoter structure diagrams for several genes involved in Fe homeostasis. White boxes indicate G-box.

Table S1. List of primers used for real-time PCR in this study

Primers	Gene	Sequences (5′-3′)
062K	*EF1α*	ATCCTGGTCAAATTGGAAACGG
063K	*EF1α*	CAGATCGCCTGTCAATCTTGG
0656	*PhbHLH121*	GCCTGAATGAACAGTTCACTG
0657	*PhbHLH121*	CAGCACTTGAATGGTATCACTG
3157	*FIT-like1*	GGTATTACGTGAAACTAGTCTG
3158	*FIT-like1*	ATGATCTGCAGAAGTAGCTAAG
0574	*bHLH038*	AATAATTCATCTCCTCCATCTCAC
0575	*bHLH038*	CTGCGCTCACTTGCGTTATG
0534	*bHLH039*	AATGTTCTGTCTTGGAGATTGTTC
0682	*bHLH039*	TCTCAAGTACATACCCGAGTTAC
0646	*BSTL*	GGAGCTTGTTGCTGTAATAAGAC
0647	*BSTL*	GAAATACATGCCTCATTAGCAAG
0640	*BTS1*	TCGCTGGATAGCTGCTCAG
0641	*BTS1*	AACTTGCTTGTCTGGATCACG
0578	*BTS2*	GAGCTGGCAGCTTCAGTTC
0579	*BTS2*	CTGTGCAGCAACCAGTGACACTAG
0650	*PYE1*	GAGTAGCTAAGACTAATCTTGAC
0651	*PYE1*	CATCTGCCTGAGATAGTTATCTG
0652	*PYE2*	CTATCTGAGCAGAATCAGCAG
0653	*PYE2*	TCCTCAGATGCTTGATCTGAG
0856	*IRT2*	CTGGCTTCATGCATGTCCTACC
0857	*IRT2*	CGTAACAACAGCAGACAACATTGC
0938	*IRT1*	GGAATCATTGTTCACTCAATAGTG
0939	*IRT1*	TGCCATTATTGCCTTCTTCCAG
0525	*FRO2*	GCCAATTACTCCAATTCTTGGA
0526	*FRO2*	CGATTGGGTAGATGTAATAACTAG
3169	*FRO1*	CGTAAGAACTATATGGTTCAAGC
3170	*FRO1*	GAAGGAAGATGATATGATAGCAC
1106	*FRO3*	CACAACTTGAGCTACAAATTGAG
1107	*FRO3*	GGTTTGAACCATATAGTCGTC
3155	*FRO4*	AGAGCAGCTCTCAATATGCTG
3156	*FRO4*	CATGTCATTGTTCTTAATCTGCAC
3167	*FRO5*	TGACAGTGTCACTTCATGCTG
3168	*FRO5*	GAGCTCAATCCATGTCACTATC
0612	*NAS1*	GCGTTGGTTGGTATGGATATC

0613	*NAS1*	GCACCATGTGCACTCCTTAG
3145	*NAS2*	GAGCAACCCTGATGCTCAG
3146	*NAS2*	CCTCATCTATTGGATGATACACC
3163	*FER1*	ATGGTACCTATTACTCCACAAAC
3164	*FER1*	TGTTGTCCATCAAGATTGATCTG
0664	*FER2*	GAGGACATGCTGAGAAATTGATG
0665	*FER2*	ACAAACTTACCATACAACGCATC
0622	*FER3*	CTTGTACCTACTGTTCCACAAG
0623	*FER3*	AAACATCGCGTGATAAACATACG
3147	*VTL2*	AGATGATGTAGAAGATGAGAAG
3148	*VTL2*	CAGCCAATAATGGCACCATTG
0632	*YSL1*	GTTCCTCTCCCAATGGCTATG
0633	*YSL1*	ATCAAACTAGCCTTCCTGCTG
0636	*YSL2*	CACAGCCATTGCCATAGGCAG
0637	*YSL2*	TGCTTATGGTTCTGGTTTGACTG
0602	*NEET*	AGCTAAAGTTGTGGACTCTGTTG
0603	*NEET*	TCACATGGCTTCCATCACATAG
3149	*ZIF1*	GGGTCACCCTCTTTGTGGC
3150	*ZIF1*	CTGGTCCACTGCTCTGTTCTG
3151	*ZIF2*	GTTGGTGAAGTACTATCGATCTC
3152	*ZIF2*	CAACTCGAGCAGTGATGATAG
3153	*ZIF3*	GCATTAGCCGCAACAGTAATC
3154	*ZIF3*	TCACGGAGATCACTGCGATC

Table S2. List of primers used for plasmids construction

Primers	Sequences (5′-3′)
35S:PhbHLH121-GFP	
0754	gagaacacgggggactctagaATGGATGAATTAAAGTGCAAG
0761	cgcccttgctcaccatggatccAGGGACGCCAGCTCTAGCCTG
35S:GFP-PhbHLH121	
0898	GGACGAGCTGTACAAGGGTCTAGAcATGGATGAATTAAAGTGCAAG
0899	GAGCTCTATCGATCAATCAGGATCCTTAAGGGACGCCAGCTCTAGC
35S:PhbHLH34-RFP	
1005	gagaacacgggggactctagaATGGTTTCACCGGAGAACACTA
1006	ctcggaggaggccatggatccAGCAACTGGAGGGCGGAGCAC
35S:PhbHLH104-RFP	
1014	gagaacacgggggactctagaATGGCGGCGGCAGAAGAAGATG
1015	ctcggaggaggccatggatccAGCAACTGGGGGACGGAGAG
35S:PhbHLH105-RFP	
1009	gagaacacgggggactctagaATGAATCAATTGGATTCAATTC
1010	ctcggaggaggccatggatccAGCAGCAGGAGGCCTGAGCTC
35S:PhbHLH115-RFP	
1019	gagaacacgggggactctagaATGGTTTCACCGGAGAACACTAA
1020	ctcggaggaggccatggatccAGCAACTGGAGGGCGGAGCAC
AD-PhbHLH34	
0984	CCATGGAGGCCAGTGAATTCATGGTTTCACCGGAGAACACTA
0985	CGATTCATCTGCAGCTCGAGTTAAGCAACTGGAGGGCGGAG
AD-PhbHLH104	
1012	ccatggaggccagtgaattcATGGCGGCGGCAGAAGAAGATG
1013	cgattcatctgcagctcgagTTAAGCAACTGGGGGACGGAG
AD-PhbHLH105	
1007	CCATGGAGGCCAGTGAATTCATGAATCAATTGGATTCAATTC
1008	CGATTCATCTGCAGCTCGAGTCAAGCAGCAGGAGGCCTGAG
AD-PhbHLH115	
1021	ccatggaggccagtgaattcATGGTTTCACCGGAGAACACTAA
1022	cgattcatctgcagctcgagTTAAGCAACTGGAGGGCGGAGC
BD-PhbHLH121	
0974	ATCTCAGAGGAGGACCTGCATATGATGGATGAATTAAAGTGCAAG
0975	TCGACGGATCCCCGGGAATTCTTAAGGGACGCCAGCTCTAGC
BD-PhbHLH121m	
0974	ATCTCAGAGGAGGACCTGCATATGATGGATGAATTAAAGTGCAAG
0979	TCGACGGATCCCCGGGAATTCTTACAATTTCTCCCGATCGGCCTTC

proGbox:HIS	
1049	aattcCACGTGCACGTGCACGTGgagct
1050	cCACGTGCACGTGCACGTGg
proPhbHLH039:LUC	
3141	gtcgacggtatcgataagcttTGGTTAGGGTTTCTAAATGTGG
3142	ccgctctagaactagtggatccTTGTTAAAACAGTGTTGTCATGTC
